# Nrf2 Activation as a Therapeutic Target for Flavonoids in Aging-Related Osteoporosis

**DOI:** 10.3390/nu17020267

**Published:** 2025-01-13

**Authors:** Samia S. Messeha, Fidara F. Fidudusola, Sherif Gendy, Lekan M. Latinwo, Caroline O. Odewumi, Karam F. A. Soliman

**Affiliations:** 1 College of Science and Technology, Florida A&M University, Tallahassee, FL 32307, USA; samia.messeha@famu.edu (S.S.M.); fidala1.fidudusola@famu.edu (F.F.F.); lekan.latinwo@famu.edu (L.M.L.); 2 College of Pharmacy and Pharmaceutical Sciences, Institute of Public Health, Florida A&M University, Tallahassee, FL 32307, USA; 3 School of Allied Health Sciences, Florida A&M University, Tallahassee, FL 32307, USA; sherif.gendy@famu.edu

**Keywords:** osteoporosis, bone, Nrf2, oxidative stress, menopause, natural products

## Abstract

Biological aging is a substantial change that leads to different diseases, including osteoporosis (OP), a condition involved in loss of bone density, deterioration of bone structure, and increased fracture risk. In old people, there is a natural decline in bone mineral density (BMD), exacerbated by hormonal changes, particularly during menopause, and it continues in the early postmenopausal years. During this transition time, hormonal alterations are linked to elevated oxidative stress (OS) and decreased antioxidant defenses, leading to a significant increase in OP. Aging is significantly associated with an abnormal ratio of oxidant/antioxidant and modified nuclear factor erythroid-derived two related factor2 (Nrf2)/Kelch-like ECH-associated protein 1 (Keap1) pathway. OS adversely affects bone health by promoting osteoclastic (bone resorbing) activity and impairing osteoblastic (bone-forming cells). Nrf2 is critical in controlling OS and various cellular processes. The expression of Nrf2 is linked to multiple age-related diseases, including OP, and Nrf2 deficiency leads to unbalanced bone formation/resorption and a consequent decline in bone mass. Various drugs are available for treating OP; however, long-term uses of these medicines are implicated in diverse illnesses such as cancer, cardiovascular, and stroke. At the same time, multiple categories of natural products, in particular flavonoids, were proposed as safe alternatives with antioxidant activity and substantial anti-osteoporotic effects.

## 1. Introduction

Osteoporosis (OP), the most prevalent comorbidity disease, is significantly associated with physiological aging [1]. This skeletal disorder is the primary reason for various complications, including bone fragility, fracture, disability, deterioration in life quality, and an increase in mortality rate [2,3]. As a woman progresses through menopause, the ovaries produce less estrogen, and these variations in estrogen exposure have a considerable influence on the majority of bodily tissues [4] and the lead of OP [5]. OP is one of the challenges associated with the onset of menopause and continues to increase in approximately 30–50% of all women in the postmenopausal phase [6,7]. Comorbidities are a common phenomenon among the elderly; in particular, OP is positioned as the most frequent [1]. Numerous modifications occur throughout aging, including the significant decline in osteoblast division and accelerated osteoblast and osteocyte apoptosis [8,9], elevated osteoblast aging [10], impaired osteoprogenitors [11], and increased bone marrow adipogenesis [12]. Indeed, the aged population is characterized by the inverse correlation between bone mass and bone marrow adipose tissue [13]. Even though age is the primary cause of oxidative stress (OS), other contributors, including lifestyle choices, diabetes, hypothyroidism, and prolonged chemotherapy treatment, are also considered risk factors for this skeleton impairment. At age fifty, 20% of males and 50% of women are expected to experience an osteoporotic fracture (OF) [14]. Nevertheless, the most frequent fracture among elderly people is a hip fracture, concurrent with an increased mortality rate [15].

OP is an advanced bone disorder that leads to diminished bone mass, proceeding to increased bone fragility, fractures, and death [2]. Two criteria, osteoblastogenesis, and osteoclastogenesis, are involved in OP disorders, and OP occurs when the osteoclastogenesis mechanism rate exceeds osteoblastogenesis [16]. Altered epigenetics has emerged as a crucial mechanism in the pathophysiology of OP [17,18,19], along with numerous hematopoietic and immune mediators in the bone microenvironment [20,21]. In aged people, many epigenetic changes are used as markers of OP [13], whereas DNA methylation is posited as the main epigenetic alteration [19,22]. DNA methylation can be modulated through physical exercise [23,24] and demethylating agents [25,26]. However, long-term exposure to these agents is frequently associated with severe side effects [27]. Furthermore, skeletal aging and OP are strongly linked to inheritable mutated genes [28], such as OPG, osteocalcin (OCN), SOST, OSX, RUNX2, RANKL, and Wnt10b [29].

## 2. Bone Composition and Metabolism

**Figure 1:**
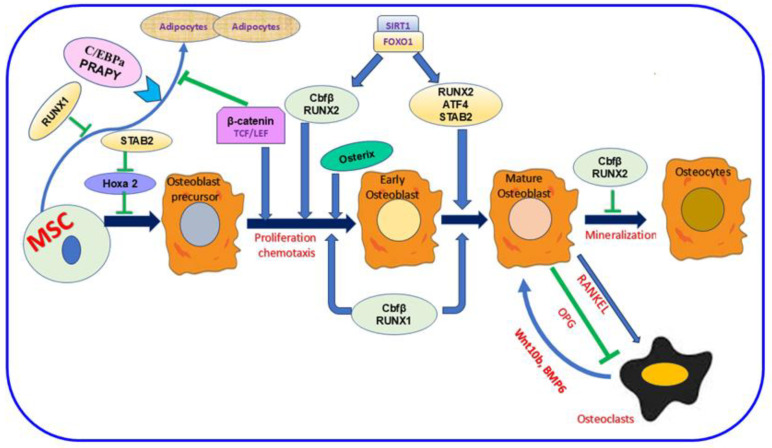
The importance of mesenchymal stem cells (MSC) in the bone-formation mechanism. The figure showed the dual role of Runx2 during bone formation. This protein and Runx1 and their co-factor Cbfβ allow osteoblast differentiation. Meanwhile, Runx2 hinders the mechanism mediating osteoclast differentiation. SIRT1 and FOXO1 are crucial proteins for enhancing Runx2 expression. Various protein codings are also involved in the different stages of differentiation as follows: Osterix and β-catenin during early osteoblast formation, SATB2, and ATF4 during osteoblast maturation. In contrast, SATB2 attenuates Hoxa2 activation in the initial stage of osteoblast formation. Osteoblasts’ and osteoclasts’ interaction is vital. Meanwhile, the RANKL signaling pathway activates osteoclast differentiation, and OPG inhibits this process. In parallel, osteoclasts can also trigger osteoblast differentiation via Wnt10b, BMP6. Runx1; Runt-related 1, Runx2; Runt-related 2, Cbfβ; Core-Binding Factor Subunit Beta, Sirtuin 1; FOXO1; Forkhead Box O1, SATB2; SATB Homeobox 2, ATF4; Activating Transcription Factor 4, Hoxa2; Homeobox A2, RANKL; Receptor Activator Of Nuclear Factor Kappa-B Ligand, OPG; Osteoprotegerin, Wnt10b; Wnt Family Member 10B, BMP6; Bone Morphogenetic Protein 6. 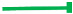; inhibition.

The bone comprises minerals, inorganic ions, proteins, fat, and other cellular elements [13,30,31]. This composition is essential to enhance bone function, keep the body’s structure, and shield the interior organs [13]. Four distinct cell kinds are identified in bone: osteocytes, osteoblasts, osteoclasts, and bone-lining cells [32,33]. These cell types are categorized based on their origin, abundance, location, and designated function [32,34]. In the bone, osteocyte cells are the most inner. Meanwhile, the other three cells are close to the bone surface [32,34]. Osteoblasts, the bone-making cells, are derived from mesenchymal stem cells (MSC) and are crucial in the bone-forming process [35,36]. Various proteins are involved in this mechanism, as shown in Figure 1. Inactive osteoblasts, bone-lining cells (BLC) [37] controlling bone resorption, osteoclast differentiation, regulating the synthesis of RANK, the receptor activator of nuclear factor kappa-B ligand, and osteoprotegerin (OPG) [38,39]. Osteocyte type with prevalent number and life span [40,41] orchestrate bone remodeling [42] and bone resorption [43,44,45,46]. Osteoclasts, the type of bone cell originating from the mononuclear hematopoietic stem cell, control bone resorption [16,47]. Increased osteoclast activity and production in OP and other bone illnesses promote unbalanced bone resorption/formation, decreasing bone density and increasing bone fracture risk [48,49]. Various proteins mediate bone formation, such as Runx1 and Runx2, and their major co-factor, Cbfβ. SIRT1 and FOXO1 also enhance the expression of Runx2 (Figure 1). The development of an osteoblast precursor into an early osteoblast is also promoted by Osterix and β-catenin; then, it progresses to a mature osteoblast in the presence of SATB2 and ATF4. In contrast, SATB2 slows Hoxa2 activity and the successive initial stages of osteoblast differentiation. RANKL is essential signaling during osteoclast differentiation; however, OPG inhibits this mechanism [36].

## 3. Bone Remodeling/Metabolism and OS

During human life, bone metabolism is controlled by bone modeling and remodeling [50,51]. In the bone modeling stage, the preformed bone transforms into a strong new bone to cope with outside factors. The remodeling mechanism maintains bone homeostasis by substituting old bone tissue with new tissue [52]. Osteoblasts and osteocytes cells are involved in the remodeling mechanism [53]. As we already know, osteocytes trigger the release of RANKL, which stimulates osteoclast activation [54]. Subsequently, the developed osteoclasts and osteoblasts form a new bone to replace the old, diminished bone [52,54,55]. Bone metabolism is also accomplished through various receptors on osteoblasts’ surface, such as estrogen, vitamin D, and other hormone receptors [54]. The multifaceted OS–bone metabolism link demonstrates the mechanism underlying OP etiology. OS has been shown to adversely affect bone health by promoting osteoclastic activity and hindering osteoblastic differentiation, initiating a net loss of bone density. Elevated reactive oxygen species (ROS) are the lead of OS [56]. Consequently, ROS accumulation enhances proteins implicated in osteoclast differentiation and activation, such as RANKL and TRAP (tartrate-resistant acid phosphatase) [57,58]. Increased ROS also leads to an elevated level of lipid peroxidation products, including malondialdehyde (MDA), which promote bone reabsorption by lowering osteoprotegerin (OPG), the typical inhibitor of osteoclastogenesis [59]. OS has been implicated in OP-linked postmenopausal complications [56].

## 4. Menopause, OS and OP

Menopause is a typical stage of aging for women [60], characterized by diminished ovarian follicular function, leading to the cessation of the menstrual cycle [61]. The number of postmenopausal women is increasing significantly with the increased life span, predicted to reach 1.2 billion by 2030 worldwide [62]. Under normal physiological conditions, natural menopause is expected in most women in their 50s. As an exception, premature or early menopause could be found in women who are 40–45 years old due to various medical interventions such as surgery or cancer therapy, leading to estrogen deficiency and increased risk for morbidity and mortality [63]. Menopause causes the alteration of sex hormones, such as the anti-Müllerian hormone, estrogen, the follicle-stimulating hormone (FSH), and insulin-like growth factors (ILGF-I) [64].

**Figure 2:**
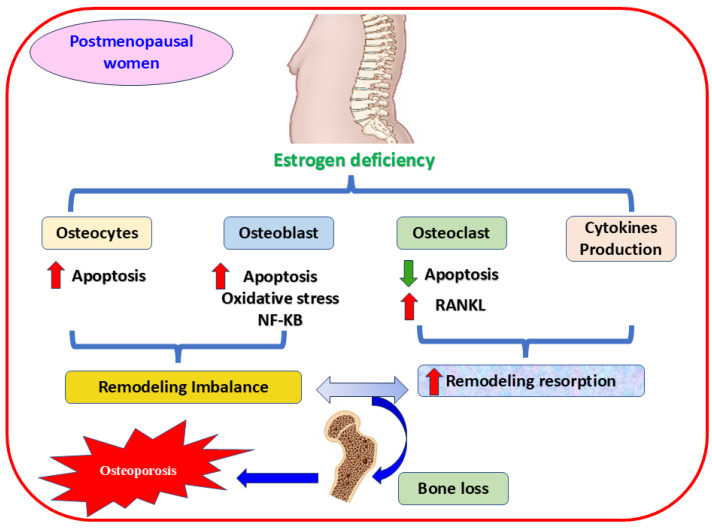
The mechanism of estrogen deficiency-mediating OP in postmenopausal women. The figure shows that estrogen deficiency induces apoptosis and activates oxidative stress, the mechanism implicated in osteoporosis, by decreasing the proliferation of osteocytes and osteoblast cells. Oxidative stress and NF-κB signaling pathways are also involved in this mechanism. In contrast, activating RANKL accelerates osteoclast proliferation, and releasing proinflammatory cytokines are vital contributors in mediating bone resorption and fragility. RANKL, receptor activator of nuclear factor kappa-B ligand; NF-κB, nuclear factor kappa-B P65 Subunit, 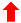 increases, 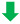 decreases.

During menopause, estrogen levels decline significantly, increasing OS, bone resorption, and OP [4,5]. Estrogen and its receptors *α* and *β* sustain bone homeostasis [65], controlling bone resorption and inhibiting apoptosis in osteoblasts and osteocytes [66]. On the contrary, estrogen hindrances osteoclast differentiation [67,68] through various mechanisms, such as attenuating the expression of multiple cytokines [69] and osteoclast-linked RANKL [70], or augmenting the release of OPG, the crucial receptor of RANKL [71]. Furthermore, a deteriorated antioxidant enzymes level in osteoporotic postmenopausal women is linked to estrogen deficiency and bone loss [72,73]. This reduction in estrogen level impacts cytokine expression and leads to inflammation and elevated ROS production, subsequent excessive bone resorption, and osteoporotic fracture [74] (Figure 2). Consequently, regulating the expression of crucial genes and triggering essential proteins have become practical approaches to managing health complications in the aged population [75].

## 5. Nrf2 and OS

**Figure 3:**
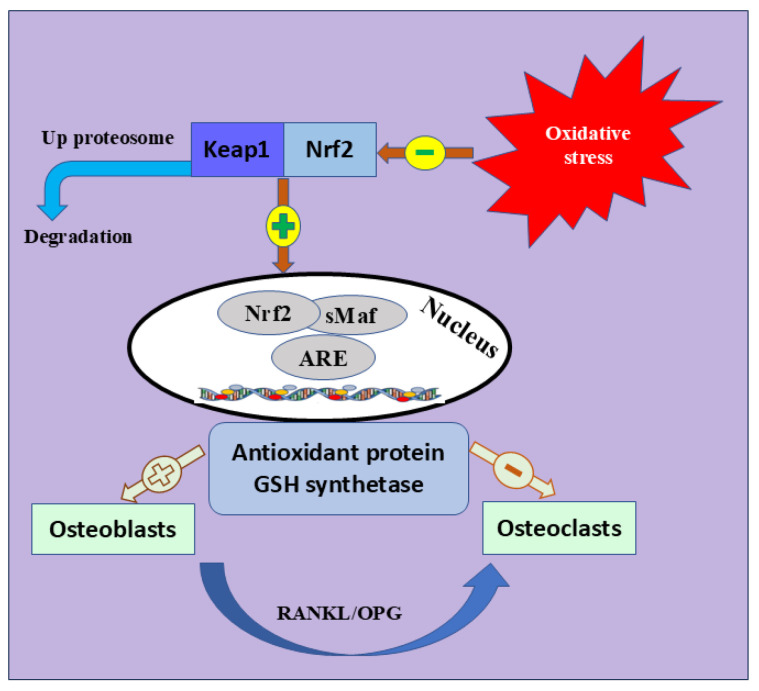
Nrf2 signaling pathway and bone formation. The coexistence of Keap1 and Nrf2 inhibits the expression of Nrf2. Physiological changes in elderly and postmenopausal women trigger OS, which leads to Keap1 inhibition and segregation of Nrf2 into the nucleus to trigger different antioxidant-mediated genes. The figure also shows the positive and negative role of antioxidant protein and GSH synthetase osteoblast or osteoclast differentiation. Keap1; Kelch Like ECH Associated Protein 1, Nrf2; Nuclear Factor Erythroid 2-Related Factor 2, GSH; Glutathione peroxidase, sMaf; Small Maf, ARE; antioxidant response element, RANKL; receptor activator of nuclear factor kappa-B ligand, OPG; OPG; Osteoprotegerin.

Nrf2 is the most significant intracellular defense mechanism that maintains a balance between oxidant production and elimination and mitigates OS [76,77]. Nrf2 belongs to the Cap ‘n’ Collar (CNC) subfamily [78]. It is characterized by seven functional protein homology domains (Nrf2-ECH homology, Neh). The different structures of Neh1-7 are a key factor in orchestrating the initiation, stability, and transcription activation of Nrf2 [79]. Many signaling pathways are also enhancing Nrf2 activation, including phosphatidylinositol 3-kinase (PI3K)/Ak strain transforming (Akt) pathway, epigenetics [80], mitogen-activated protein kinase (MAPK), and c-Jun *N*-terminal kinase (JNK) signaling pathway [81]. Meanwhile, Keap1 is crucial in halting the transcriptional activation of Nrf2 [82,83]. During ordinary oxidative contexts, Nrf2 is localized in the cytosol; however, its endogenous suppressor, Keap1, lowers its expression [84,85]. Once activated by OS, Nrf2 disconnects from Keap1, moves to the nucleus, and activates many endogenous antioxidants [86,87], such as glutathione sulfhydryl transferases (GSTs), catalytic enzymes of glutathione (Glutathione peroxidase, GSH-Px), peroxidase (PRDXs), superoxide dismutase (SOD), and catalase (CAT) [88]. These antioxidants mediate inflammation, OS, and identification of DNA damage [89,90] (Figure 3).

## 6. Nrf2 Role in OP During Menopause

**Figure 4:**
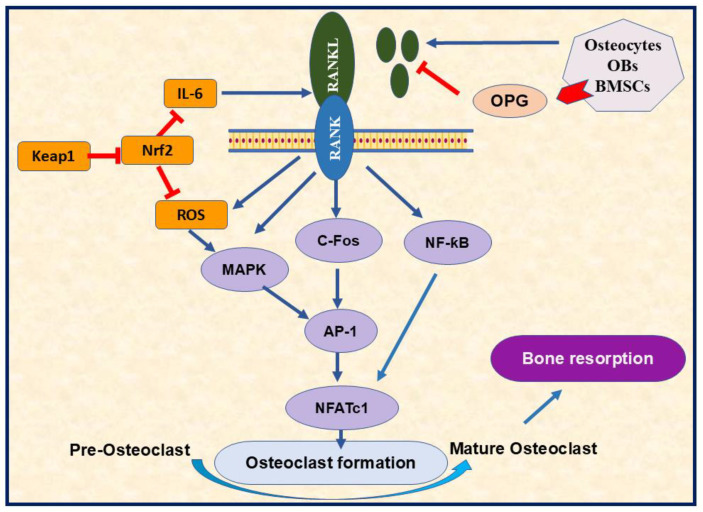
The importance of RANK/RANKL signaling pathway in osteoclasts differentiation. The RANK/RANKL pathway regulates various signaling involved in osteoclastogenesis, such as MAPK, cFos, and NF-κꞵ through AP-1 and NFATc1 activation. RANK/RANKL also triggers ROS production, the mechanism that enhances osteoclastogenesis. The diagram highlighted the role of Nrf2 in decreasing osteoclastogenesis by targeting ROS formation. Furthermore, OPG can inhibit osteoclast formation by targeting RANK-RANKL binding. RANK; receptor activator of nuclear factor kappa B 2, RANKL; receptor activator of nuclear factor kappa-B ligand, OBs; osteoblasts, BMSCs; bone marrow stromal cells, OPG; osteoprotegerin, Nrf2; nuclear factor erythroid 2-related factor 2, Keap1; kelch like ECH associated protein 1, ROS; reactive oxygen species, IL-6; interleukin 6, MAPK; mitogen-activated protein kinase, *C*-Fos; cellular Fos proto-oncogene; NF-κB; nuclear factor kappa-B P65 Subunit, AP-1; activator protein 1, NFATc1; nuclear factor of activated T cells 1, 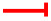 Inhibition.

The elaborate connection between Nrf2 and menopause mainly revolves around OS and fluctuating hormones during and after the menopausal transition [91]. Inactive Nrf2 is expressed in most cells, including osteoclasts, osteoblasts, and osteocytes. Nrf2 deficiency promoted RANKL-induced osteoclast differentiation, bone resorption, and MAPK activation [92] (Figure 4). Previous research highlighted the influence of estrogen on Nrf2 activation through genomic and non-genomic avenues, the mechanism that boosts the body’s antioxidant defenses [93]. In contrast, the reduced estrogen level in postmenopausal women is implicated in decreased Nrf2 expression, increased OS markers, and imbalanced redox homeostasis, significantly targeting bone [94]. Therefore, manipulating Nrf2 expression through hormone replacement administration (HRT) may help to relieve some menopausal symptoms associated with OS [95].

## 7. Nrf2 and OS Role in OP

The Nrf2 signaling pathway is a fundamental mediator in orchestrating OS-linked bone homeostasis [52,96]. There is a significant association between OS and OP. Cardiovascular diseases, diabetes, and smoking are risk factors for OP that are linked to OS elevation [97,98]. ROS-mediated OS can seriously impair bone homeostasis and skeletal fragility [99,100]. ROS accumulation is a significant cause of bone loss among old people. Meanwhile, Nrf2 maintains bone homeostasis by induction of distinct genes-linked OS [101,102]. Nrf2 deficiency-mediated OS is previously demonstrated in OP complications [103], increased radiation-triggered bone fragility [104], impaired bone homeostasis, decreased bone density and strength [96], in addition to the rise in intracellular ROS levels, the mediator of bone fragility [92,104,105]. In contrast, inducing Nrf2 expression is a protective approach against OP [106,107]. Hence, attenuating the Nrf2 pathway could also initiate and enhance OP progression [108]. In osteoblasts and osteoclasts, controlling Keap1-facilitated Nrf2 activation is a critical mechanism for maintaining bone homeostasis [109]. Interestingly, antioxidant injections protected ovariectomized rats from OS via Nrf2 activation-accompanying diminished ROS concentration. Together, they support the concept that Nrf2 upregulation is an anticipated approach for maintaining bone homeostasis and managing OP [109,110].

## 8. Medication-Induced OP

Primary OP is an age or postmenopausal-related disease. Meanwhile, various causes can lead to secondary OP, such as hypothyroidism, diabetes, and chronic treatment with synthetic glucocorticoids [111]. Unfortunately, these drugs lead to glucocorticoid-related OP and osteonecrosis [112,113] due to increased OS, ROS, and prospective mitochondrial membrane damage [114,115,116]. Furthermore, some drugs might limit bone resorption but do not restore bone mass, ultimately decreasing bone turnover [116]. As previously mentioned, OS-related diseases downregulate Nrf2 [117], causing ROS accumulation that triggers GLOP development [118]. This mechanism emphasizes the importance of Nrf2 activation as a suggested therapeutic key for GLOP [119]. Furthermore, the degradation of superoxide dismutase (SOD) by ROS leads to substantial bone loss [73,103,110,120]. These findings highlight the crucial function of Nrf2 in osteoprotection [107].

## 9. The Importance of the Nrf2 Signaling Pathway in Fracture Healing

**Figure 5:**
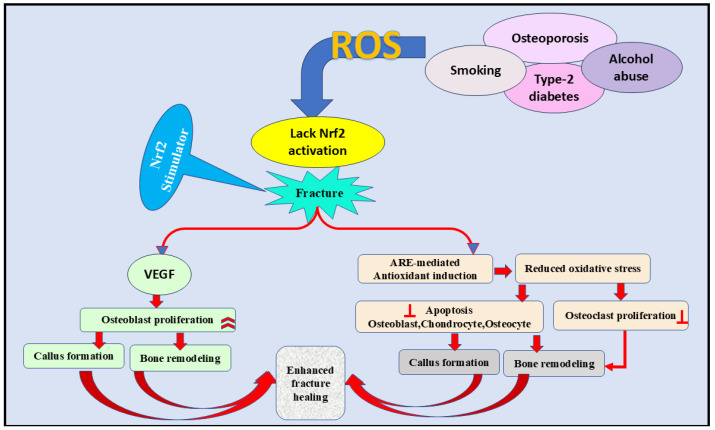
The role of the Nrf2 signaling pathway in the fracture healing process by inhibiting ROS formation. Furthermore, VEGF activation and antioxidant induction are essential contributors to fracture healing through decreasing oxidative stress, increasing osteoblast proliferation, and decreasing osteoclast proliferation. VEGF; vascular endothelial growth factor, ARE; antioxidant-rsponse element, ROS; reactive oxygen species, 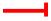 inhibition.

Elevated expression of Nrf2 during fracture healing [121] demonstrates the significant role of this signaling pathway in managing bone formation [52]. This concept was evidenced by the slow bone-healing mechanism in Nrf2 defective mice compared with the normal [121]. In this study, the inhibition of vascular endothelial growth was the underlying process mediating the delayed bone healing [121]. Likewise, Nrf2 was also found to stimulate stem cells during tissue renewal [109]. In contrast, ROS obstruct bone homeostasis and fracture healing by stimulating osteoclasts, pausing the differentiation of osteoblasts, osteocytes, and chondrocytes [58,122,123,124,125,126]. Nrf2 signaling can also enhance fracture healing (Figure 5) by triggering antioxidant production and protecting bone cells from ROS-induced complications [109].

## 10. Therapeutic Approaches for OP Treatment

Many approaches have been introduced to manage OP, such as diagnosis, fracture risk assessment, and antiresorptive anabolic drugs. The available antiresorptive agents include anti-RANKL antibodies, hormone-substitution therapy, bisphosphonates, raloxifene, and estrogen-receptor mitigators. Anabolic agents can accelerate new bone formation and stimulate bone density at low doses [127,128]. Unfortunately, various studies revealed the link between these drugs and the manifestation of serious illnesses such as stroke, cancer, and cardiovascular disorders [127,129]. Consequently, finding an alternative safe treatment is highly demanded. Natural compounds with minor side effects have been used in treating different diseases, including bone diseases. They could be projected as an alternate medicine to manage the anticipated side effects of traditional drugs [130].

## 11. The Possible Mechanisms of Flavonoids in Managing OP

Flavonoids are a distinguished group of natural compounds that we consume daily in food, including vegetables, fruits, cereal, wine, and tea [131,132]. Modern technology has boosted the extraction of these naturally found flavonoid compounds [133,134]. Flavonoids can be classified into three main sub-groups: iso-flavonoids (phytoestrogens), bioflavonoids, and neoflavanoids [135]. Nonetheless, these sub-groups have diverse chemical and biological properties [136]. These natural flavonoids, remarkably found in pharmaceutical manufacturing, are suggested as future therapeutic mediators in various domains [137]. Flavonoids have been used as anticancer, antioxidant, anti-inflammatory, antiresorptive, antiviral, free radical scavengers, in addition to its significant role in treating cardiovascular disorders [137,138]. Flavonoid is an alternative drug for treating OP to overcome the serious side effects associated with traditional hormone therapy [139]. These compounds have been used in treating bone loss and fracture-linked postmenopausal OP [140,141,142]. Other flavonoids such as quercetin, daidzein, kaempferol, and genistein are extensively examined. Interestingly, most investigated flavonoids have shown antioxidant properties or increased osteoblast proliferation but decreased osteoclast differentiation, and substantial anti-osteoporotic effects [47]. Soy isoflavones also revealed significant antiresorptive activity by slowing osteoclasts and advocating osteoblast differentiation [143].

Quercetin is a naturally found flavonoid in vegetables and fruits, and it has antioxidant and bone-conserving properties [130]. This flavonoid possesses several mechanisms that enhance bone, including the inhibiting of RANKL-mediated osteoclast differentiation [144] and regulating MAPK and Nrf2 signaling pathways, which are involved in osteoblast production and osteoclast differentiation [130]. Quercetin also activates the ERK signal pathway, advocating the segregation of Nrf2 from Keap1 [145] to suppress the expression of NF-κB [146]. Furthermore, quercetin augments various genes mediating the Nrf2 pathway [132,147].

Genistein is an iso-flavonoid phytoestrogen found in soybeans and exhibiting estrogenic and anti-estrogenic properties [148,149,150]. These characteristics enhance bone mineral density (BMD) in postmenopausal women with osteopenia [151]. Genistein utilizes different mechanisms to maintain bone homeostasis, such as promoting osteoblastic differentiation, suppressing tumor necrosis factor-alpha (TNF-α), the osteogenesis inhibitor [152], and preventing osteoclastogenesis and OP [153]. Furthermore, the compound upregulates ERK1/2 and protein kinase C (PKC) signaling pathways to upregulate Nrf2 mRNA and protein expression and protect cells against OS.

Kaempferol and its byproducts are generously obtained from various vegetables and fruits. Kaempferol has controlled numerous diseases, including bone disorders [154]. Earlier studies have advocated the significant role of this compound in bone formation by increasing osteogenesis while decreasing adipogenesis [155,156]. Kaempferol attenuated the TNF-α-mediate NF-κB pathway and RANKL-induced differentiation of osteoclasts [157,158,159]. Kaempferol activates the Nrf2/ARE pathway by upregulating the ERK and MAPK signaling pathways, and WNT/β-catenin [81,160,161].

Myricetin, one of the flavanol subclass, is obtained from many therapeutic plants, vegetables, fruits, and tea [162,163]. Myricetin has shown several effects, including its ability to protect osteogenesis and inhibit osteoclast differentiation [164]. Myricetin controls osteoclast differentiation and bone resorption by targeting the RANK/RANKL pathway to repress osteoclastogenic markers, inhibiting the common proinflammatory cytokines and inflammatory mediators, mainly TNF-α to suppress the NF-κB pathway [165]. Interestingly, the mechanism of NF-κB repression is facilitated by Nrf2/HO-1 upregulation [166].

The flavonoid icariin is used to moderate various bone diseases such as OP [167] by inhibiting RANKL-involved osteoclast differentiation and suppressing bone resorption by initiating osteoclast apoptosis [168]. Icariin also prevents RANKL-induced osteoclastogenesis and bone resorption by targeting MAPK and NF-κB signaling pathways [169]. This flavonoid also utilizes various mechanisms to increase osteoblasts’ mineralization and proliferation rate and maintain bone homeostasis [170]. Indeed, icariin triggers the Nrf2 signaling pathway to stimulate the antioxidative stress activity and inhibit NF-κB [171], upregulating glutathione through the PI3K/Akt/Nrf2 pathway [172].

Luteolin is also a flavonoid extracted from various herbs and used in pharmaceutical industries [173]. As exhibited by other flavonoids, luteolin has shown the ability to prevent bone loss by modulating bone resorption, inhibiting RANKL-mediated osteoclastogenesis, and eliminating OS markers [174,175], or inducing PI3K-AKT [176]. Further properties were also revealed by luteolin, such as the ability to induce the osteoblastic process and enhance collagen production [177], inhibit essential proinflammatory cytokines, and target ROS production [178].

Hesperidin belongs to the flavanones subgroup, and it is the main compound in citrus fruits [179]. This flavone has a substantial advantage in bone health [180]. Hesperidin was also revealed to stimulate Nrf2 [81] by endorsing PI3K/AKT and Wnt/b-catenin signaling pathways [80,181] and to inhibit bone resorption and NF-κB-triggered osteoclastogenesis [182]. This compound was also found to reduce OS, inflammation, p53, and manage estrogen pathway [183,184,185].

Apigenin is a member of the subgroup flavone, generously found in various vegetables and fruits [173]. This compound has a significant role in preventing bone loss [186,187,188,189,190]. Apigenin upregulates glutathione peroxidase and antioxidant enzymes to decrease ROS production. Furthermore, this flavone augments the expression of many genes mediating osteoblast differentiation, while IL-6 and NO were abolished [191]. Some signaling pathways, such as JNK, p38 MAPK, and NF-κB signaling pathways, play a crucial role during apigenin-triggered osteogenesis, demonstrating the advantages of apigenin in managing OP [191,192].

The isoflavonoid daidzein is a phytoestrogen that is found mainly in soy products. Hence, this compound has been introduced as an alternative to estrogen replacement therapy [193,194,195,196]. Daizen boosts the growth of osteoblast cells [197] and increases the expression of crucial osteogenesis genes [198]. Furthermore, daidzein increased RUNX2 expression and OPG production and reduced RANKL [199]. A substantial reduction in NF-κB and the osteoclastogenesis inducers ROS and TNF-α was also revealed in daidzein-treated OVAX mice [200,201].

Other natural flavonoids, such as capsaicin, were previously found to improve OP in OVX rats [202]. Capsaicin increases bone maturity, BMD, femoral trabecular area, and calcium and estrogen levels while reducing TNF-α and alkaline phosphatase (ALP) [202]. Fisetin, a constituent of *Rhus succedanea,* was previously found to prevent bone resorption and osteoclast differentiation. Fisetin prevents RANKL-involved ROS production by triggering Nrf2-linked induction of antioxidative enzymes, including HO-1, glutathione-S-transferase (GST), NQO-1, and glutamate-cysteine ligase (GCL) [203]. Alpinumisoflavone, derived from *Derris eriocarpa*, has considerably moderated glucocorticoid-induced OP in animal studies. This property was exhibited by inhibiting ROS level, whereas activating the Nrf2 pathway and its downstream molecules NQO-1 and HO-1, the mechanism that reverses the osteoporotic process of glucocorticoid. Neobavaisoflavone, a Chinese plant *Psoralea corylifolia derivative*, demonstrated an anti-osteoporotic effect in OVAX mice by inhibiting osteoclastogenesis and reducing bone loss [204,205]. This compound also protects osteoblasts against dexamethasone-generated OS by stimulating Nrf2/HO-1/NQO1 signaling pathway [206]. In consistency, the natural compounds anthocyanins have shown antioxidant properties by increasing Nrf2/HO-1 expression, whereas the ROS level was inhibited [207].

## 12. Clinical Trials

Meta-analyses of animal studies typically encourage more human clinical trials [208]. Flavonoids have shown a profound effect on bone health. However, only a limited number of studies have expanded beyond animal models [143]. For example, treating people with quercetin at 0.5 g/day for three months enhanced their bone health [130]. In an analogous trial, postmenopausal women who received icariin, genistein, and daidzein at a combination dosage for 24 months recognized decreased bone loss and increased BMD [140]. Many clinical trials have investigated the prospective of isoflavones to enhance (BMD). These studies have revealed the ability of soy isoflavones to lower bone loss at the lumbar spine [209,210,211]. Furthermore, the ability of genistein to manage bone metabolism in postmenopausal patients was revealed in a previous cohort study [212]. Another clinical trial also proposed curcumin as a promising natural compound for inhibiting bone loss [213]. At the same time, further human clinical trials are necessary due to the limitations of animal models in assessing the therapeutic effectiveness of icariin in treating bone diseases [140].

## 13. Conclusions

OP is a significant health complication among the senior community. The progression of this bone disorder progresses to fractures. This implication, accompanied by limited mobility, is a substantial financial burden on the health care provider. Declined antioxidant enzyme levels in osteoporotic postmenopausal women are implicated in estrogen deficiency and bone loss. Long-term exposure to antiresorptive and/or anabolic agents, the traditional treatment for OP, is associated with severe health complications [130]. Therefore, finding an alternative medicine is highly demanded to limit the use of these drugs. Natural flavonoids have shown promise as a safe treatment choice in various applications, most notably in pharmaceutical and health care. Of many, natural flavonoids are characterized by antioxidant properties that propose these compounds as safe compounds for managing many diseases. As demonstrated by previous investigations, these natural drugs have the potential to boost bone formation and reduce bone resorption. Additionally, new research has illuminated the significance of the Nrf2 signaling pathway, which has become a key policy in controlling OS-mediated bone homeostasis. Thus, it is relevant to consider that Nrf2 overexpression may be a valuable approach for sustaining bone health and treating various skeleton illnesses, such as OP and the corresponding bone fracture [109].

## Data Availability

No new data were generated or analyzed during this study.
